# Rectal metastasis from primary small-cell lung cancer: A case report and literature review

**DOI:** 10.1097/MD.0000000000048604

**Published:** 2026-05-08

**Authors:** Weiyu Zhai, Shubiao Chen, Zhongchao Wang

**Affiliations:** a Department of Oncology, Xinyi People’s Hospital, Xuzhou, Jiangsu Province, China.

**Keywords:** case report, Rectal metastases, small cell lung cancer

## Abstract

**Rationale:**

Small-cell lung cancer (SCLC) is a subtype of primary lung cancer that has a very poor overall survival rate because of its propensity for metastasis and incredibly high proliferation rate. For most patients with lung cancer, metastatic disease is discovered at the time of initial diagnosis. About 70% of individuals with SCLC already have metastases in their brain, liver, lymph nodes, and bones at the time of diagnosis. Rectal metastases are uncommon.

**Patient concerns:**

In this case report, we described a 73-year-old female patient who was diagnosed with neuroendocrine SCLC based on a lung biopsy obtained by needle. The patient had blood in the stool, accompanied by loss of appetite and pain around the umbilicus for 6 months. The symptoms gradually worsened.

**Diagnoses:**

Rectal pathological results revealed that metastatic poorly differentiated neuroendocrine carcinoma originated from the lung.

**Interventions:**

The patient underwent 4 courses of chemotherapy.

**Outcomes:**

The hematochezia then subsided, and radiological evaluation of the rectal metastases and primary lung tumors suggested a partial response.

**Lessons:**

When a rectal tumor is identified, it is important to differentiate metastatic diseases and make a definitive diagnosis through detailed immunohistochemical evaluation and systemic imaging surveillance.

## 1. Introduction

Small-cell lung cancer (SCLC), one of the most common malignant tumors, accounts for 15% to 17% of all lung cancers.^[1]^ Rapid growth, high mortality, early metastases to distant areas, and low survival rates are its defining characteristics. The 5-year survival rate of extensive-stage SCLC is only 1% to 2%.^[2]^ For most patients with lung cancer, metastatic disease is discovered at the time of initial diagnosis. About 70% of individuals with SCLC already have metastases in their brain, liver, lymph nodes, and bones at the time of diagnosis. Intestinal metastases of lung cancer are very rare. Only 0.3% to 1.7% of patients with lung cancer had gastrointestinal metastases, and ~4.6% to 14% of patients with lung cancer developed gastrointestinal metastases during their autopsy after their death.^[3]^ When a rectal tumor is identified, it is important to differentiate metastatic diseases. Because the treatment methods and prognosis for primary rectal cancer and metastatic rectal cancer are ifferent. Colorectal metastases of lung cancer are often exposed tumors and can be diagnosed using endoscopic forceps biopsy. Endoscopic ultrasound-guided fine needle biopsy (EUS-FNB) may be informative in cases of a non-exposed rectal tumor.

In this case report, we described a 73-year-old female patient who had a lung biopsy diagnosis of neuroendocrine SCLC, and electronic colonoscopy suggested rectal metastasis from SCLC. Due to the rapid growth, high mortality, early metastases to distant areas, and low survival rates of SCLC,early diagnosis and early treatment is particularly important.

### 1.1. Patient information

A 73-year-old female patient had blood in the stool, accompanied with loss of appetitemn and pain around the umbilicus for 6 months. The symptoms gradually worsened. She was previously healthy, with no underlying medical conditions.

### 1.2. Clinical findings

The patient’s lungs had clear breath sounds and no dry or wet rales were heard. The abdomen is soft. There is positive tenderness around the umbilicus, and no rebound tenderness. There is no edema in both lower extremities.

### 1.3. Timeline

The patient began experiencing bloody stools, loss of appetite, and abdominal pain around August 2023. These symptoms persisted, progressively worsened, and led to a medical visit in February 2024 due to aggravated abdominal pain.

### 1.4. Diagnostic assessment

**Figure 1.**
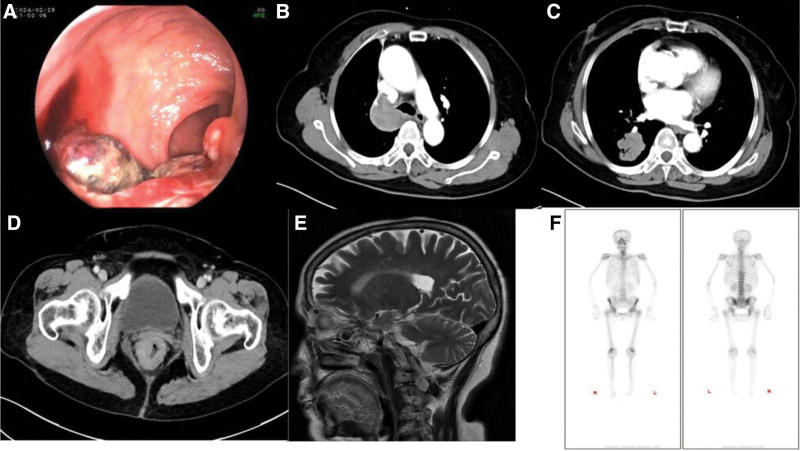
Electronic rectoscopy showed rectal tumor (A). Enhanced chest computed tomography (CT) scan revealed the neoplasms in the right upper lobe with a mediastinal lymph node metastasis (B, C). Enhanced abdominal CT showed rectal tumor (D). Cerebral magnetic resonance imaging (MRI) and bone emission CT (ECT) revealed no metastasis (E, F). CT = computed tomography, ECT = emission computed tomography, MRI = magnetic resonance imaging.

**Figure 2.**
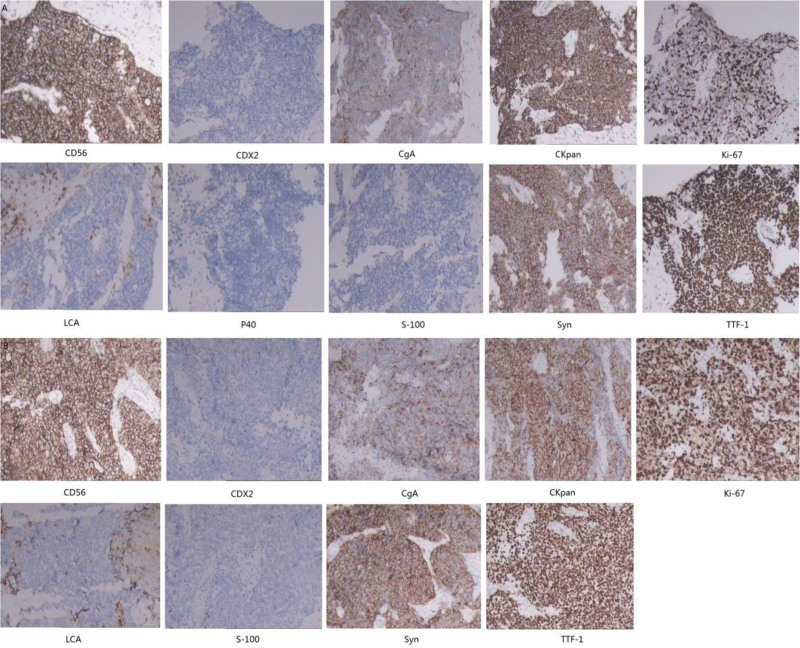
**S**mall-cell carcinoma of the right lung immunohistochemistry showed that CKpan, TTF-1, Syn, CgA, and CD56 were positive. LCA, P40, CDX2, and S-100 were negative. Ki67 was 60%+ (A). Rectal carcinoma immunohistochemistry showed that CKpan, TTF-1, Syn, CgA, and CD56 were positive. LCA, P40, CDX2, and S-100 were negative. Ki67 was 90%+ positive (B). CD56 = cluster of differentiation 56, CDX2 = caudal type homeobox 2, CgA = chromogranin A, CKpan = pan cytokeratin, Ki67 = Ki-67 antigen, LCA = leukocyte common antigen, P40 = P40 protein, S-100 = S100 protein, Syn = synaptophysin, TTF-1 = thyroid transcription factor-1.

Electronic colonoscopy showed a rectal tumor (Fig. 1A). The colonoscopy revealed a ulcerative lesion ~5 cm from the anal opening, occupying about 2/5 of the lumen, with a narrowed cavity. The lesion could be visualized through the scope and there was bleeding upon touching. Enhanced computed tomography (CT) scan revealed that the neoplasms were located in the right upper lobe near the hilum and right lower lobe. The right upper lobe showed atelectasis, and the lymph nodes under the tracheal bulge were considered metastatic (Fig. 1B, C). The lower rectal mass was considered to be rectal cancer with multiple lymph node metastasis (Fig. 1D). Cerebral magnetic resonance imaging and bone emission CT revealed no metastasis (Fig. 1E, F). CT-guided percutaneous lung biopsy was performed in March 01, 2024. The pathological findings suggested small-cell carcinoma of the right lung. Immunohistochemistry showed that pan cytokeratin, thyroid transcription factor-1, synaptophysin, chromogranin A,and cluster of differentiation 56 were positive (Fig. 2A). However, leukocyte common antigen, P40 protein, caudal type homeobox 2, and S100 protein were negative. Ki-67 antigen (Ki67) was 60%+. Rectal pathological results revealed that metastatic poorly differentiated neuroendocrine carcinoma originated from the lung. Immunohistochemistry showed that pan cytokeratin, thyroid transcription factor-1, synaptophysin, chromogranin A, and cluster of differentiation 56 were positive (Fig. 2B). Leukocyte common antigen, P40 protein, caudal type homeobox 2, and S100 protein were negative. Ki67 was 90%+ positive. The immunohistochemical results of the 2 groups were the same except for Ki67,which was higher in the rectal group. Based on the above findings, a diagnosis of small cell lung cancer with rectal metastasis was confirmed. The clinical stage is stage IV.

### 1.5. Therapeutic intervention

The patient was started on systemic chemotherapy with etoposide and lobaplatin. She tolerated chemotherapy well. The patient underwent 4 courses of chemotherapy, then the hematochezia had subsided.

### 1.6. Follow-up and outcomes

**Figure 3.**
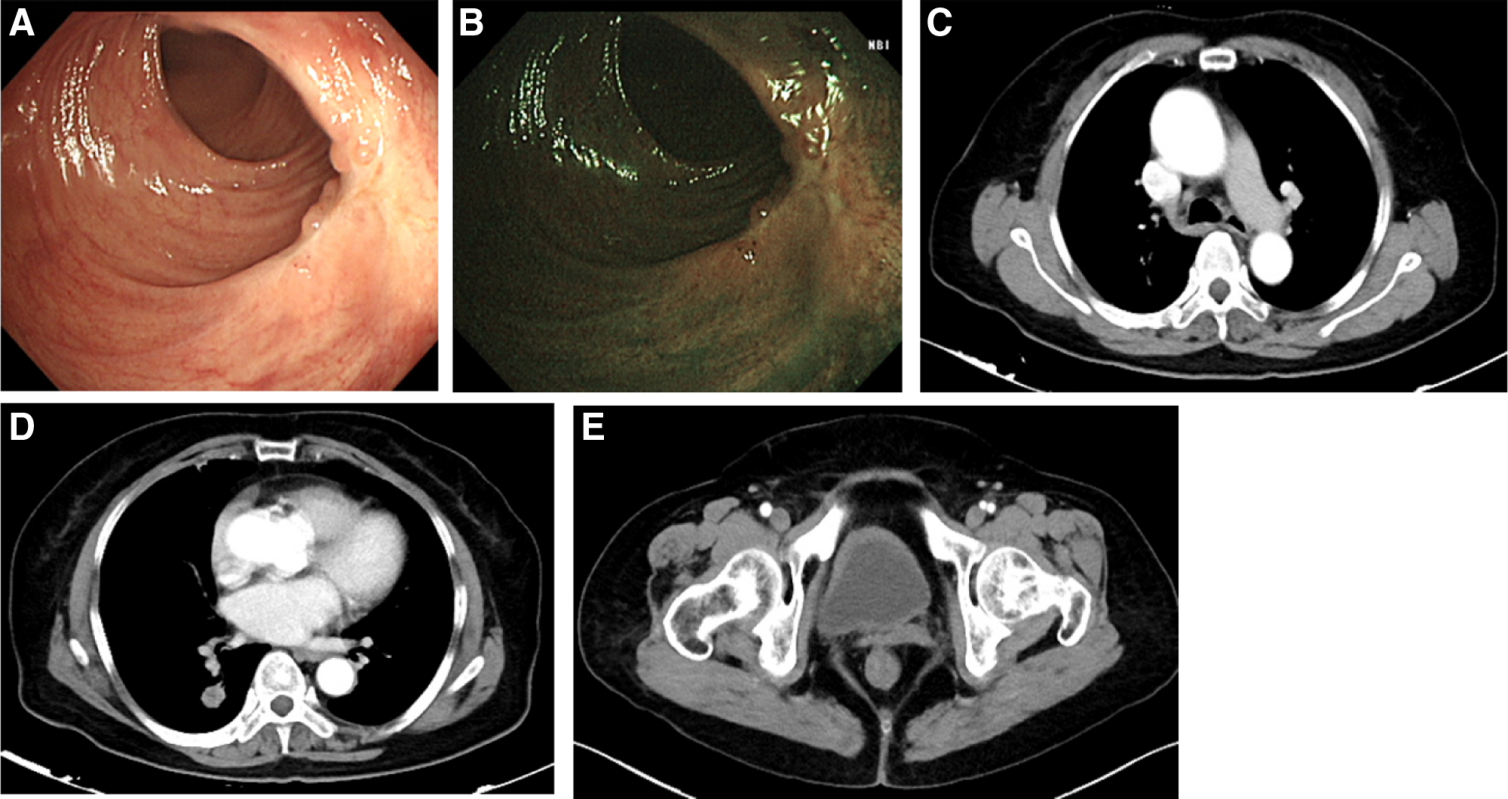
Electronic proctoscopy showed a significant reduction of rectal tumors than before treatment (A, B). CT scan revealed the neoplasms in the right upper lobe and rectal tumor shrank after treatment (C–E). CT = computed tomography.

Radiological evaluation of rectal metastases and primary lung tumors suggested a partial response (Fig. 3A–E). Serum neuron-specific enolase and pro-gastrin-releasing peptide decreased from 32 ng/mL, and 563 pg/mL to 13.5 ng/mL and 32.2 pg/mL, respectively. The patient did not experience any significant side effects.

## 2. Discussion

SCLC is a highly malignant subtype of lung cancer. In the initial diagnosis of SCLC, inoperability and metastasis are the leading causes of death and treatment failure.^[4,5]^ Lymph nodes, liver, brain, and bone metastasis are common sites of SCLC, and about 70% of patients have metastases at the time of diagnosis.^[1]^ However, rectal metastasis from SCLC is very rare. The incidence of colonic metastasis is only 0.1%.^[6]^ Most gastrointestinal metastases of lung cancers are asymptomatic, with only 0.2% to 0.5% of patients presenting symptoms.^[7]^ Shinji Mukawa had report a patient with hyperintestinal peristalsis and diarrhea. He was diagnosed with rectal metastasis from lung cancer,based on immunohistochemical analysis by EUS-FNB and image findings.^[8]^

SCLC has many clinical features. It has been classified into 4 subtypes based on the expression of different transcription factors. About 80% groups of all SCLC exhibit high expression levels of neuronal differentiation 1 and achaete-scute family bHLH transcription factor 1. The high expression of neuroendocrine genes is called as neuroendocrine SCLC. By contrast, POU class 2 homeobox 3 and yes-associated protein 1 do not express the neuroendocrine gene panel,so they are called as non-neuroendocrine SCLC.^[9]^ A short survival period is correlated with high neuron-specific enolase levels in patients with SCLC.^[10]^ At present, the standard treatment for SCLC remains to be a combination of etoposide and platinum, and there is no definitive treatment plan for the above mentioned subtypes of SCLC. Unlike the cases of Shinji Mukawa and Rui Nian,^[11]^ this case achieved a good therapeutic effect with chemotherapy alone, without undergoing colostomy, local radiotherapy or immune checkpoint inhibitor therapy.

Distant metastasis is the major cause of treatment failure in SCLC, and the underlying mechanisms remain unclear. The more common organs with distant metastases in patients with SCLC are bone (28.6%), brain (23.8%), liver (15.0%), and contralateral lung (11.7%).^[12]^ Axon-like protrusions contribute to SCLC metastasis. Loss of axon-like processes inhibits the migration of SCLC cells. Knockdown genes of protrusion formation reduces metastatic capacity.^[13]^ Besides, achaete-scute family bHLH transcription factor 1 and neuronal differentiation 1 are essential genes for the metastasis of some SCLCs.^[14,15]^ Another significant study suggested that amplification and/or over expression of nuclear factor I B can promote metastasis in a mouse model of SCLC.^[16]^ Moreover, lysine methyltransferase 2C deficiency promotes SCLC metastasis through DNA methyltransferase 3A-mediated epigenetic reprogramming.^[17]^ Loss of expression of natural killer group 2 member D ligand due to histone deacetylation is a mechanism of the deregulated innate recognition in SCLC.^[18]^ The long non-codingRNAs ITPR1 antisense RNA 1 promotes SCLC metastasis by facilitating P21 HRAS splicing and stabilizing DEAD-box polypeptide 3 to activate the rapidly accelerated fibrosarcoma-mitogen-activatedprotein kinase/ERK kinase-extracellular signal-regulated kinase cascade.^[19]^ Recently, carboplatin and etoposide together with anti-PD1 or anti-PD L1 antibody have been approved for first-line treatment for metastatic SCLC.^[18]^ This patient did not test programmed cell death-ligand 1 expression level and did not receive immunotherapy because of financial constraints. In the LEAD study, low-dose radiation plus durvalumab and etoposide/platinum in ES-SCLC prolonged the median progression free survival.^[20]^ Radiotherapy played an important role in the radical and palliative treatment of SCLC. For patients with extensive-stage SCLC who respond well to first-line treatment, chest radiotherapy or prophylactic craniocerebral irradiation may be used to delay recurrence and metastasis and prolong survival. Moreover, radiotherapy may effectively relieve the symptoms of tumor complications such as symptomatic brain metastasis, superior vena cava syndrome, rectal metastasis, bone metastasis, and malignant spinal cord compression. If the condition improves after treatment, we might consider local radiotherapy for lung lesions and metastases.

Although the mechanisms of SCLC metastasis vary, its underlying mechanisms still need further investigation. New approaches to limit the metastatic spread of tumor cells and target metastasis therapy are critical to the successful treatment of SCLC.

In conclusion, we report a case of SCLC presenting with a rectal metastasis. Rectal metastasis should be considered when patients have rectal symptoms and a history of primary lung cancer. Intestinal tract investigation should be performed for early detection and treatment. Endoscopic forceps biopsy and EUS-FNB may be informative in a rectal tumor. Ultimately, early detection and therapy have been thought to improve survival.

## Acknowledgments

We certify that there is no conflict of interest with any financial organization regarding the material discussed in the manuscript.

## Author contributions

**Formal analysis:** Weiyu Zhai.

**Investigation:** Weiyu Zhai, Shubiao Chen.

**Supervision:** Weiyu Zhai.

**Visualization:** Zhongchao Wang.

**Writing – original draft:** Shubiao Chen.

**Writing – review & editing:** Zhongchao Wang.
